# A Convenient and Sensitive LC-MS/MS Method for Simultaneous Determination of Carbadox- and Olaquindox-Related Residues in Swine Muscle and Liver Tissues

**DOI:** 10.1155/2018/2834049

**Published:** 2018-06-14

**Authors:** Heying Zhang, Wei Qu, Yanfei Tao, Dongmei Chen, Shuyu Xie, Lingli Huang, Zhenli Liu, Yuanhu Pan, Zonghui Yuan

**Affiliations:** ^1^MOA Laboratory for Risk Assessment of Quality and Safety of Livestock and Poultry Products, Huazhong Agricultural University, Wuhan, Hubei 430070, China; ^2^National Reference Laboratory of Veterinary Drug Residues and MAO Key Laboratory for Detection of Veterinary Drug Residues, Huazhong Agricultural University, Wuhan, Hubei 430070, China

## Abstract

This paper presents a convenient and sensitive LC-MS/MS method for the simultaneous determination of carbadox and olaquindox residues, including desoxyolaquindox (DOLQ), desoxycarbadox (DCBX), quinoxaline-2-carboxylic acid (QCA), 3-methyl-quinoxaline-2-carboxylic acid (MQCA), and the glycine conjugates of QCA and MQCA (namely, QCA-glycine and MQCA-glycine, resp.) in swine muscle and liver tissues. Tissue samples were extracted with 2% metaphosphoric acid in 20% methanol and cleaned up by solid-phase extraction (SPE) on a mixed-mode anion-exchange column (Oasis MAX). Analysis was performed on a C_18_ column by detection with mass spectrometry in the multiple reaction monitoring (MRM) mode. The limits of detection (LODs) of the six analytes were determined to be 0.01 *μ*g·kg^−1^ to 0.25 *μ*g·kg^−1^, and the limits of quantification (LOQs) were 0.02 *μ*g·kg^−1^ to 0.5 *μ*g·kg^−1^. The total recoveries of the six analytes in all tissues were higher than 79.1% with the RSD% less than 9.2%. The developed method can determine the real residue level of QCA and MQCA, whether they are present in free form or as glycine conjugates in tissues, together with the carcinogenic desoxy metabolites DCBX and DOLQ with high recovery. Therefore, this method was suitable for routine analysis of residue control programmes and the residue depletion study of CBX and OLQ on swine.

## 1. Introduction

Carbadox (CBX) and olaquindox (OLQ) have been used as antimicrobial drugs in the feed of swine for growth promotion and the increased rate of weight gain and to control swine dysentery and bacterial enteritis in young swine [1]. Since 1998, both drugs have been withdrawn from market in the European Union due to possible carcinogenic and mutagenic effects of the drugs and their desoxy metabolites [2–5]. Nowadays, CBX is still permitted to use in the USA and several countries, and OLQ is permitted in China as a premix used in swine feed for growth promotion. Metabolism studies have revealed that OLQ was *in vivo* transformed into desoxyolaquindox (DOLQ) by the reduction of the *N*-oxide, and the latter could be further hydrolyzed to 3-methyl-quinoxaline-2-carboxylic acid (MQCA). More than 80% of the extracted residues in the liver and kidney tissues of swine were DOLQ, and the second metabolite that accounts for 13% of the total residue in the liver was identified as a carboxylic acid derivative (named 2-carboxymethylaminocarbonyl-3-methylquinoxaline, identical to the structure of the glycine conjugate of MQCA) [6]. The metabolism of CBX was characterized by the rapid reduction of the *N*-oxide groups to give desoxycarbadox (DCBX) and the cleavage of the methyl carbazate side chain to give the carboxaldehyde which was further oxidated to the corresponding carboxylic acid, quinoxaline-2-carboxylic acid (QCA). The major urinary metabolite was shown to be the QCA, which was present in a free form and as its glycine conjugate [7]. Since 1991, QCA and MQCA have been designated as the residue markers for CBX and OLQ, respectively, and maximum residue limits (MRLs) of 30 and 5 *μ*g·kg^−1^ in the liver and muscle tissues of pigs were recommended [8, 9]. However, at its 60th meeting in 2003, the Committee reported that QCA is not a suitable marker for monitoring carcinogenic metabolites of CBX, and QCA does not ensure the absence of carcinogenic residues in the liver and muscle tissues [10]. To ensure the safety of porcine products concerning the two drugs and especially to guarantee carcinogenic-free metabolites in the tissues of food-producing animals, many methods have been developed including HPLC-UV [11], GC-MS [12, 13], LC-MS [8, 14–16], and LC-MS/MS [8, 17, 18]. However, these methods usually focus on the determination of MQCA and QCA, sometimes including DCBX; moreover, the sample pretreatment involved either alkaline hydrolysis or protease digestion (or digestion with the enzymes) in order to release the conjugated or protein-binding “marker residue,” followed by liquid-liquid extraction and cleanup with solid-phase extraction (SPE), which are relatively laborious and time-consuming and usually lead to low recovery especially for DCBX [8].

Since no analytical methods to quantify the main metabolites of CBX and OLQ were reported yet, the aim of this work was to develop a sensitive and reliable LC-MS/MS method for simultaneous determination of the carcinogenic desoxy metabolites DOLQ and DCBX and the marker residues QCA and MQCA (presented in free form or as glycine conjugates, namely, QCA-glycine and MQCA-glycine, resp.) in swine muscle and liver tissues.

## 2. Experimental

### 2.1. Chemicals and Reagents

**Scheme 1:**
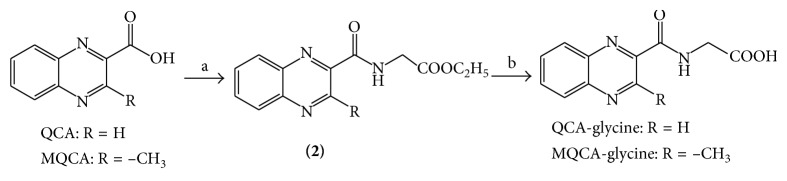
Synthesis route of QCA-glycine and MQCA-glycine. Reagents and conditions: (a) glycine ethyl ester, DCC, DMAP, and DCM at room temperature for 4 h; (b) (1) 1 M NaOH at room temperature for 2 h and (2) HCl.

Standards for DCBX, DOLQ, and MQCA were obtained from the Institute of Veterinary Pharmaceuticals (Huazhong Agricultural University, Wuhan, China). QCA was purchased from Sigma-Aldrich (St. Louis, MO, USA). The glycine conjugations of MQCA and QCA were prepared using a common method as illustrated in Scheme 1. Briefly, MQCA (or QCA), DCC, glycine ethyl ester, and a catalytic amount of DMAP were stirred at room temperature for 4 hours. The urea was filtered off, the solvent was evaporated in a vacuum, and the intermediate (**2**) was obtained. Then, (**2**) was hydrolyzed with 1 M sodium hydroxide to give the desired compound MQCA-glycine (or QCA-glycine). After recrystallizing from methanol for 3 times, QCA-glycine and MQCA-glycine with purity above 98% were obtained. Distilled water was further purified by passing through a Milli-Q Integral water purification system (Millipore, Bedford, USA). HPLC-grade methanol and acetonitrile were purchased from Tedia (Fairfield, OH, USA).

The stock standard solutions of all analytes (1 mg·mL^−1^) were prepared by exactly weighing the necessary quantities of the substances and dissolving each in methanol. All stock solutions of the individual substances were stored in amber vials at −20°C and were stable for 6 months. Working combined mixed standard solutions (1.0 *μ*g·g^−1^) were prepared by diluting the stock standards in methanol. The working standard mixture solutions were stable for at least 3 months for all substances when stored in amber vials below 4°C.

### 2.2. LC-MS/MS Analysis

The LC-MS system (Shimadzu Co., Ltd.) was composed of an autosampler (SIL-20AC), a solvent delivery pump (LC-20AD), and a column oven (CTO-20AC) with an API5000 triple-quadrupole instrument (Applied Biosystems, Foster City, CA); Biosystems Sciex Analyst software version 1.5 was used to process data. Chromatographic separation was performed on a Thermo Scientific Hypersil Gold C_18_ column (150 mm × 2.1 mm i.d.; 5 *μ*m particles) using a gradient elution consisting of mobile phase A (0.1% formic acid in water) and mobile phase B (acetonitrile). The flow rate was 0.2 mL/min with linear gradient at the following conditions: 0–12 min 85% to 20% A, 12–17 min 20% A, and 17–17.5 min return to 85% A. The injection volume was 10 *μ*L. The source/gas conditions were as follows: the curtain gas was set at 6 psi, while the ion source gas 1 (GS1) and ion source gas 2 (GS2) were set at 60 psi and 50 psi, respectively. The compound conditions were entrance potential 10.0 V and collision cell exit potential 2.0 V. The mass spectrometer was operated in a multiple reaction monitoring (MRM) mode that selected one precursor ion and two suitable product ions for each target compound. The parameters of the *m*/*z* and collision energy of parent ions and quantitative product ions of DCBX, DOLQ, QCA, MQCA, QCA-glycine, and MQCA-glycine are shown in Table 1.

### 2.3. Sample Extraction and Cleanup

Aliquots of 2.0 ± 0.1 g of homogenized tissue samples were placed into a 50 mL centrifugal tube. 5 mL of 5% (w/v) metaphosphoric acid in 20% (v/v) methanol was added into the samples, and the mixtures were sonicated in an ultrasonic bath at room temperature for 10 min. After centrifugation at 8000 rpm for 10 min, the supernatants were transferred to a new tube, and the residue was extracted again as described above. The extracted solutions were merged, and 1 mol·L^−1^ sodium acetate was added to adjust the pH to about 7. The extracts were ready for further SPE cleanup.

The Oasis MAX cartridge column (60 mg, 3 mL) (Waters Corp., Milford, MA, USA) was conditioned sequentially with 3 mL of methanol and 3 mL of water. The filtered extract was loaded onto the column at a flow rate of 1 mL·min^−1^. The cartridge was washed with 30 mL sodium acetate/methanol (95 : 5, v/v) and then dried by purging air at a rate of 10 mL·min^−1^ for 5 min. The analytes were eluted with 15 mL 2% trifluoroacetic acid in methanol at a flow rate of 1.0 mL·min^−1^. The eluent was evaporated to dryness under a stream of nitrogen at 40°C and reconstituted in acetonitrile-water solution (15 : 85 (v/v), 200 *μ*L) for LC-MS/MS analysis.

### 2.4. Validation Study

The analytical method developed for determination of the residues of CBX and OLQ and their main metabolites in swine muscle and liver tissues was validated according to the EU Decision 2002/657/EC [19]. The selectivity, matrix effects, linearity, CC*α*, CC*β*, accuracy, and precision of the method were evaluated by spiking the six reference compounds into the blank matrices.

#### 2.4.1. Selectivity

The selectivity of the method was evaluated by duplicate analysis of 10 blank samples of swine muscle and liver tissues. The blank samples were spiked with the six analytes (LOQ level) and processed by the proposed extraction method. The chromatograms of blank samples were compared and analyzed with those of spiked plasma samples.

#### 2.4.2. Linearity and Matrix Effects

Linearity was evaluated from the calibration curves by triplicate analysis of blank tissue samples fortified with the analytes at five concentration levels (DCBX, DOLQ, QCA-glycine, and MQCA-glycine at 0.05, 0.10, 0.2, 0.5, and 5 *μ*g·kg^−1^; QCA and MQCA at 0.50, 1.0, 2.5, 5.0, and 10.0 *μ*g·kg^−1^). Linearity was expressed as the coefficient of linear correlation (*r*) and from the slope of the calibration curve. To evaluate the degree of ion suppression or signal enhancement, matrix-matched calibration curves were established. Matrix effects were assessed by comparing the slopes of these calibration curves using the following formula: matrix effect (ME) = 1 − (*a*_matrix_/*a*_standard_) × 100, where *a*_matrix_ and *a*_standard_ are the slopes of calibration straight lines for standard and matrix-matched calibration graphs.

#### 2.4.3. Detection Limits and Detection Capability

The limit of detection (LOD) was determined by successive analysis of spiked matrices with decreasing amounts of every standard until a signal-to-noise (S/N) ratio of 3 : 1 was reached. The limit of quantification (LOQ) was defined as the lowest concentration of an analyte that can be quantified with acceptable precision and accuracy.

The CC*α* and CC*β* were determined by analysis of 20 blank tissue samples, and the signal-to-noise (S/N) ratio is calculated at the time window in which the analyte is expected. The CC*α* values were calculated as three times the S/N ratio. The CC*β* was calculated by analyzing 20 blank samples spiked with concentration at CC*α*. Then, the CC*β* value was added up to 1.64 times the corresponding standard deviation.

#### 2.4.4. Accuracy and Precision

The accuracy and precision were determined in all matrices of the six analytes at three different concentrations (low, middle, and high levels) in six replicates using different analytical batches. The recovery was calculated by the following formula: (the measured level/the fortified level) × 100%. The precision of the method was determined by intraday and interday results and expressed by the relative standard deviation (RSD). Intraday precision was conducted on the same day. Interday precision was determined by repeating the study on 3 consecutive days.

## 3. Results and Discussion

### 3.1. Extraction and Cleanup Procedure

In previous studies on quantitative determination of carbadox and olaquindox residues in the porcine muscle or liver, the samples were subjected either to alkaline hydrolysis at 105°C for 1 h or protease digestion overnight to release the QCA and MQCA [11, 12]. These methods are obviously tedious and time-consuming and usually lead to low recovery and sample throughput. In addition, it has been noted that the residues of the carcinogenic and mutagenic metabolites (i.e., DCBX and DOLQ) must be monitored for the regulation of these AGPs in food animal production. Boison et al. have demonstrated that even QCA was stable under this excessive pretreatment process, and DCBX was very rapidly degraded and undetectable [8]. So, the extraction and cleanup procedure should meet the request that QCA and MQCA, whether they exist in free form or as conjugate with glycine, together with DCBX and DOLQ, could be determined with high recovery.

In this work, 2% metaphosphoric acid in 20% methanol was used as the extracting solvent and applied for deproteination to release the binding metabolites. Without further alkaline hydrolysis or protease digestion, the analytes were subjected to cleanup by SPE columns (Waters Oasis MAX). The analytes could be eluted smoothly with 2% trifluoroacetic acid in methanol. This mixed-mode anion-exchange sorbent for sample cleanup proved to be effective to decrease the matrix effect and to enhanced a good recovery, thus significantly improving the measurement precision.

### 3.2. LC-MS/MS of the Analytes

**Figure 1:**
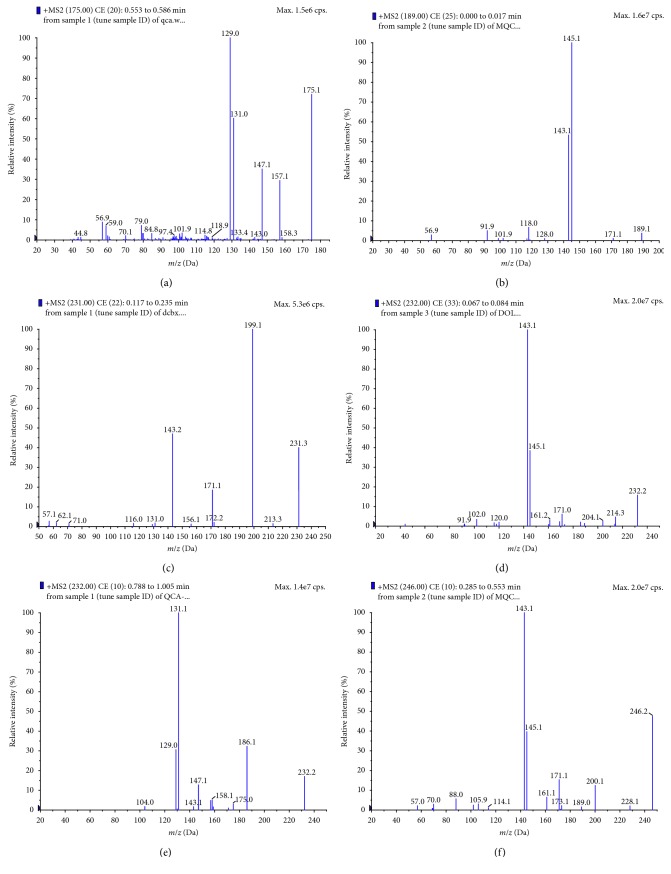
Mass spectra of (a) QCA, (b) MQCA, (c) DCBX, (d) DOLQ, (e) QCA-glycine, and (f) MQCA-glycine.

The MS/MS of QCA and MQCA exhibits a similar MS/MS fragmentation pattern which has been described previously [15, 16]. DCBX showed a molecular ion at *m*/*z* 231 amu and a prominent product ion at *m*/*z* 231 → 199 amu, resulting from *α*-fragmentation of the methyl ester to loss of OCH_3_. The second prominent product ion at *m*/*z* 143 was attributed to the successive loss of carbon monoxide and N_2_ that is supported by the observation of a moderate peak at *m*/*z* 199 → 171 amu. DOLQ showed a molecular ion at *m*/*z* 232 amu and two prominent product ions at *m*/*z* 143 and *m*/*z* 145. QCA-glycine and MQCA-glycine showed similar fragmentation patterns, which was characterized by the successive loss of water and carbon monoxide of glycine to produce the product ions at *m*/*z* 232 → 186 amu and *m*/*z* 246 → 200 amu, respectively. The second prominent product ions of them were at *m*/*z* 129 and *m*/*z* 143, just like those of QCA and MQCA, respectively. The mass spectra of MQCA, QCA, DCBX, DOLQ, QCA-glycine, and MQCA-glycine are shown in Figure 1.

### 3.3. Method Validation

**Figure 2:**
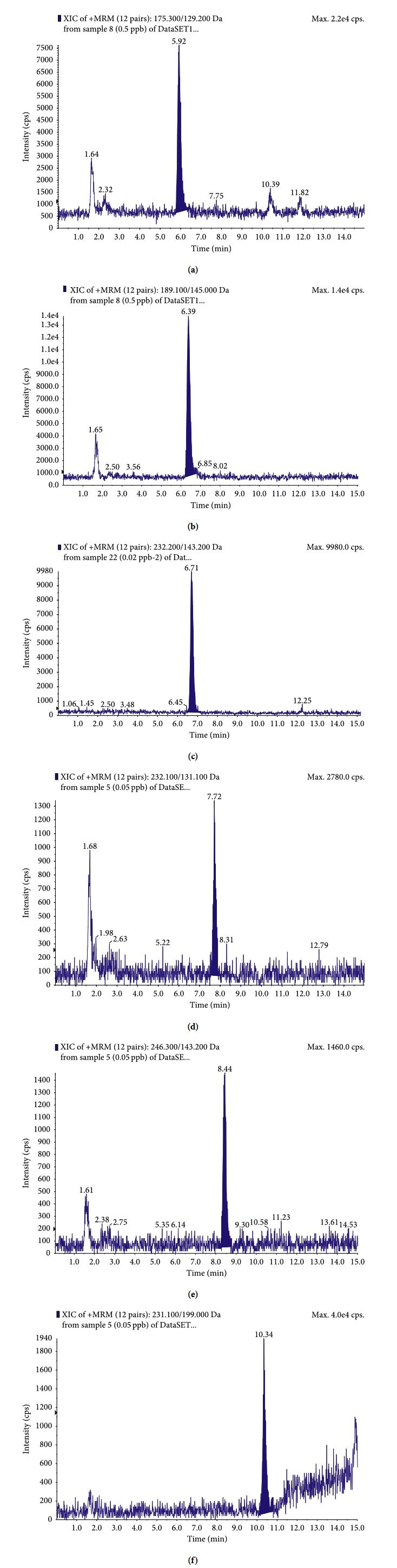
LC-ESI-MS/MS chromatograms in a positive-ion mode of (a) QCA (175.3 > 129.2), (b) MQCA (189.1 > 145.0), (c) DOLQ (232.2 > 143.2), (d) QCA-glycine (232.1 > 131.1), (e) MQCA-glycine (246.3 > 143.2), and (f) DCBX (231.1 > 199.0) fortified in the liver of swine.

Figure 2 shows the representative MRM chromatograms of blank liver samples spiked with the analytes at 0.5, 0.5, 0.05, 0.02, 0.05, and 0.05 *μ*g·kg^−1^ for QCA, MQCA, DCBX, DOLQ, QCA-glycine, and MQCA-glycine, respectively. Under the optimized condition, no significant interfering peaks were observed in chromatograms of all tested matrices near the retention time of the analyte. All the peaks of the analytes were detected to have high resolution and good peak shape.

According to the European Union Commission Decision 657/2002 [19], a minimum of 4 identification points (IPs) are required to unambiguously confirm the residues in food of animal origin. The described method monitored 2 product ion scores and 3 IPs, plus 1 IP for the precursor ion, to fulfill the criteria for confirmation of the analytes. Moreover, all measured ion ratios in unknown samples must correspond to those in standards within predefined limits. The maximum permitted tolerance in the ion ratios varies with the relative intensity of the product ions to the base peak. In this method, the occurrence of MQCA, QCA, DCBX, DOLQ, QCA-glycine, and MQCA-glycine in real samples was confirmed by comparing the ion ratios of the two MRM transitions with those obtained from the matrix-matched calibration curve standards. All of the samples used for the validation study met the relevant identification criteria (Table 2).

The linearity of the analytical response across the studied range for all the analytes was excellent, with correlation coefficients higher than 0.997. The linear dynamic range of the mass spectrometer was also estimated for all the analytes from a matrix-matched calibration curve. The matrix spike curve showed good linearity within the tested range 0.02–50 *μ*g·kg^−1^ for DOLQ, 0.05–50 *μ*g·kg^−1^ for DCBX, QCA-glycine, and MQCA-glycine, and 0.5–50 *μ*g·kg^−1^ for QCA and MQCA, respectively, and the matrix effects were found to be in a range of 11.5–23.8%. Results for the calibration curve and coefficient in the swine liver are shown in Table 3. The LOQs of QCA and MQCA were determined to be 0.5 *μ*g·kg^−1^, which were comparable to those reported in literature [8, 15]. When QCA and MQCA conjugated with glycine, the LOQs could be lowered by an order of magnitude (0.5 *μ*g·kg^−1^ versus 0.05 *μ*g·kg^−1^). The two desoxy metabolites, DCBX and DOLQ, showed high sensitivity with an LOQ of 0.05 *μ*g·kg^−1^ and 0.02 *μ*g·kg^−1^, respectively. CC*α* and CC*β* of the analytes in swine tissues ranged from 0.02 *μ*g/kg to 0.3 *μ*g/kg and 0.05 *μ*g/kg to 0.5 *μ*g/kg, respectively. The mean accuracy values obtained in the recovery tests were between 79.1% and 91.1%. In 2007, the European Union Reference Laboratory (Fougeres, France) proposed for DCBX, QCA, and MQCA in meat a recommended concentration of 10 *μ*g·kg^−1^ as a minimum requirement for the analytical method [20]. The developed method well fulfilled the requirements for the monitoring of the residues related to CBX and OLQ. The relative standard deviation (RSD) of interday values of the six compounds analyzed by the present method was 5.5–9.2% and for the intraday test was 5.0–7.4% (Table 4). The results showed that the established method was accurate and precise and fit for the purpose of CBX and OLQ residue detection in swine muscle and liver tissues.

### 3.4. Applicability of the Proposed Method

The method established above was then applied to the analysis of real samples including 50 swine muscles and 50 swine livers from different supermarkets in Wuhan (Hubei Province, China). All the samples were analyzed for QCA, MQCA, DCBX, DOLQ, QCA-glycine, and MQCA-glycine residues to determine whether there exists misuse/or illegal use of the AGPs in the locality. Eight samples out of 50 swine liver samples were found to contain DOLQ residues (the amount was between 0.65 and 2.35 *μ*g·kg^−1^), in which three samples were also found to contain MQCA residues (the amount was between 0.76 and 1.22 *μ*g·kg^−1^).

## 4. Conclusions

This paper describes a convenient and sensitive LC-MS/MS method for the simultaneous determination of residues of the metabolites of CBX and OLQ in swine muscle and liver tissues. Since there existed a doubt whether QCA and MQCA are suitable residue markers for the regulation of these two AGPs in food animal production, the residues of the carcinogenic and mutagenic metabolites (i.e., DCBX and DOLQ) should also be monitored for regulatory control. Therefore, this quantitative and confirmatory method can be used for routine analysis for regulating the use/misuse of CBX and OLQ in food animal production and for residue the depletion study of CBX and OLQ.

## Figures and Tables

**Table 1:** Optimum precursor and product ions with the respective collision energy (eV) for MS/MS.

Compound	Q1 (*m*/*z*)	Q3 (*m*/*z*)	CE (eV)	DP (V)
MQCA	189.1	145.0^a^	18	51
		101.9	35	75

QCA	175.3	129.2^a^	15	54
		101.9	38	77

DOLQ	232.2	143.2^a^	15	51
		102.0	38	85

DCBX	231.1	199.0^a^	35	95
		143.2	38	91

MQCA-glycine	246.3	200.1	10	45
		143.2^a^	35	90

QCA-glycine	232.1	186.1	10	48
		131.1^a^	32	80

^a^The most abundant ion (also used for quantification).

**Table 2:** LC-MRM parameters of MQCA, QCA, DOLQ, DCBX, MQCA-glycine, and QCA-glycine.

Analytes	Average of ion ratios of matrix-matched standard solution (RSD%)	Maximum permitted tolerances	Average of ion ratios of spiked porcine tissues (RSD%)
MQCA	0.60	0.50–0.72 (1 ± 20%)	54
QCA	0.65	0.54–0.78 (1 ± 20%)	62
DOLQ	0.45	0.36–0.54 (1 ± 25%)	38
DCBX	0.52	0.43–0.62 (1 ± 20%)	48
MQCA-glycine	0.46	0.37–0.58 (1 ± 25%)	42
QCA-glycine	0.40	0.32–0.50 (1 ± 25%)	32

**Table 3:** Calibration curves of the analytes fortified in the swine liver.

Compound	Calibration curve	Coefficient of correlation *r*	Linear range (*μ*g/kg)
QCA	*y* = 52357*x* + 12354	0.9998	0.5–50
MQCA	*y* = 99929*x* + 19199	0.9997	0.5–50
DCBX	*y* = 270179*x* + 73776	0.9998	0.05–50
QCA-glycine	*y* = 138502*x* + 58623	0.9991	0.05–50
DOLQ	*y* = 182083*x* + 61571	1	0.02–50
MQCA-glycine	*y* = 222520*x* + 55169	0.9997	0.05–50

**Table 4:** Validation parameters related to the method of QCA, MQCA, DCBX, DOLQ, QCA-glycine, and MQCA-glycine in the swine liver.

Parameter	QCA	MQCA	DCBX	DOLQ	QCA-glycine	MQCA-glycine
Transition	175.3/129.2	189.1/145.0	231.1/199.0	232.2/143.2	232.1/131.1	246.3/143.2
LOD (*μ*g/kg)	0.10	0.25	0.02	0.01	0.02	0.02
LOQ (*μ*g/kg)	0.50	0.50	0.05	0.02	0.05	0.05
CC*α* (*μ*g/kg)	0.10	0.30	0.02	0.02	0.02	0.02
CC*β* (*μ*g/kg)	1.10	1.33	0.15	0.08	0.12	0.15
Precision (RSD%)					
Intraday (*n*=5)	5.5	6.6	6.0	6.8	9.2	7.6
Interday (*n*=5)	5.0	5.5	6.4	7.3	7.1	7.4
Recovery (%)	85.6 ± 3.4	79.1 ± 6.7	85.5 ± 4.6	91.1 ± 6.3	87.3 ± 7.4	89.1 ± 8.1

## Data Availability

The data used to support the findings of this study are available from the corresponding author upon request.
